# Intestines of a Pig, Which, for Six Weeks before Death, Had Been Fed with “Typhoid Dejections”

**Published:** 1859-06

**Authors:** 


*Intestines of a Pig, which, for six weeks before death, had been fed with "Typhoid Dejections."*

Dr. Murchison exhibited these to the Pathological Society of London, and observed that although it was generally admitted that the true typhus fever is eminently contagious, many still entertained doubts as to the contagious nature of the so-called "typhoid fever;" yet it was difficult to explain in any other way, the facts which had been adduced by Bretonneau, Gendron, Piedvache, and others. Some observers, and more particularly Dr. Budd, of Bristol, and the late Dr. Snow, had thought that typhoid fever was propagated by the dejections from the bowels. Without questioning the validity of this supposition, Dr. Murchison expressed his belief that many of the facts which had been urged in its support might be explained on the hypothesis of a spontaneous origin of the fever from the putrid emanations from the drains, which had been thought merely to convey the poison. All those who had considered that the fever might be communicated by the dejections had been strong opponents of the possibility of its spontaneous origin. It was obviously of great importance, both in a medical and a sanitary point of view, to determine whether fever might be communicated in the manner just alluded to. The experiment had been undertaken in order to throw some light upon this question; and its results were offered simply for what the results of one experiment might be worth. A pig had been selected for the experiment, for the following reasons: 1. Because in its diet it approached most nearly to man; and it was thought that less difficulty would be encountered in making it submit to the experiment than with other animals. 2. There were few or no animals in which the structures that became specially diseased in typhoid fever, viz., Peyer's patches, were so well developed. 3. Because there was evidence that the pig was liable to typhoid fever. Cases of the disease, in this animal, in which the characteristic lesions had been found after death, have been described by Falke and other writers on veterinary medicine. The pig selected was between three and four months old. Care was taken that the dejections were obtained from typhoid patients in whom they presented the light ochrey color peculiar to the disease in the most marked degree; they were mixed up with barley-meal and other articles of food. The first was given on Sept. 9th, 1858. For the first three weeks one was given every day, or every second or third day. During the next fortnight, two or three were given every day; and, during the last week, one every second day. They were eaten greedily. On two different occasions, during the first fortnight, the animal had slight diarrhœa, lasting for twelve hours, and its ears felt rather hot; but these symptoms speedily subsided. With these exceptions, the animal exhibited no abnormal symptoms; its stools were of normal consistence, and it increased greatly in weight and size, as was shown by measurements taken at the commencement and at the termination of the experiment. On Oct. 23d it was killed, and its body opened. There was abundance of subcutaneous fat, and the muscular tissue appeared healthy in every respect. The intestines throughout were healthy. There was not the slightest trace of any recent or old ulceration anywhere, nor of any thickening or alteration of Peyer's patches, or of the solitary glands. The mesenteric glands were not enlarged.—[*London Lancet.*

